# Interdisziplinäres Multimodales Assessment: Interprofessionelle Interaktion in Teamsitzung und Abschlussgespräch

**DOI:** 10.1007/s00482-023-00740-7

**Published:** 2023-08-10

**Authors:** Leonie Schouten, Ulrike Kaiser, PAIN2020-Team, Thomas Isenberg, Gabriele Lindena, Carolin Martin, André Möller, Katharina Augustin, Ulrike Kaiser, Anne Gärtner, Anke Preißler, Greta Hoffmann, Julia Pritzke Michael, Frank Petzke, Michael Pfingsten, Leonie Schouten, Karin Deppe, Hans-Raimund Casser, Bernd Nagel, Katja Schwenk, Beatrice Metz-Oster, Lena Milch, Jana Rensland, Thomas Kohlmann, Daniel Szczotkowski, Ursula Marschall, Catharina Schumacher, Frank Petzke

**Affiliations:** 1 Schmerzmedizin, Klinik für Anästhesiologie, Universitätsmedizin Göttingen, Robert-Koch-Str. 40, 37075 Göttingen, Deutschland; 2 Klinik für Anästhesiologie und Intensivmedizin, Universitätsklinikum Schleswig-Holstein, Ratzeburger Allee 160, 23538 Lübeck, Deutschland

**Keywords:** Schmerzen und Risikofaktoren, Interdisziplinäre multimodale Schmerztherapie, Ambulante Diagnostik, Partizipative Forschung, Teamprozess, Pain and risk factors, Interdisciplinary multimodal pain therapy, Outpatient diagnostics, Participative research, Team process

## Abstract

**Hintergrund:**

In PAIN2020 (Innovationsfond, 01NVF17049) wurde ein frühzeitig im Krankheitsverlauf ansetzendes, ambulantes Interdisziplinäres Multimodales Assessment (IMA) eingeführt. Zentrales Qualitätsmerkmal ist die enge interdisziplinäre Zusammenarbeit der Schmerzmedizin, Physiotherapie und Psychologie, die eines komplexen Organisations- bzw. Abstimmungsprozesses, insbesondere in Teamsitzung und Abschlussgespräch, bedarf.

**Ziel:**

Die (unterschiedlichen) Sichtweisen der beteiligten Berufsgruppen werden im Teamprozess als gemeinsamer Konsens zusammengeführt. Der Ablauf zur Gestaltung der Interaktion der Berufsgruppen untereinander in Teamsitzung und Abschlussgespräch sowie mit den Patient:innen soll untersucht (qualitativ) und diskutiert werden.

**Methodik:**

In PAIN2020 fand ein Workshop zum IMA statt, um die im Prozess bisher gewonnenen Erkenntnisse und Erfahrungen durch das Monitoring mit Mitarbeitenden bzw. Teams der PAIN2020-Zentren gemeinsam zu reflektieren. In einer von drei Arbeitsphasen wurden in interprofessionell zusammengestellten Gruppen im Rahmen eines World Cafés in drei rotierenden Durchgängen Aussagen der Teilnehmenden zur Gestaltung der Interaktion in Teamsitzung und Abschlussgespräch eingeholt.

**Ergebnisse:**

Es konnten förderliche und hinderliche Faktoren für die Gestaltung interdisziplinärer Zusammenarbeit in Teamsitzung und Abschlussgespräch identifiziert werden, die übergeordnet in einem Rahmenmodell zusammengeführt wurden.

**Diskussion:**

Das Erbringen der neuen Versorgungsleistung als eine interdisziplinäre Aufgabe im Team geht über bestehende Struktur- und Prozessparameter in der Definition von Rahmenbedingungen in der Interdisziplinären Multimodalen Schmerztherapie hinaus und sollte daher ebenfalls personale Kompetenzen und Fachkompetenzen mitberücksichtigen. Für die Umsetzung des IMAs ergeben sich daher neue Dimensionen, die zukünftig diskutiert werden sollten.

## Abstract (en)

**Background:**

In PAIN2020 (Innovation Fund, 01NVF17049), an outpatient interdisciplinary multimodal assessment (IMA) was introduced early in the course of the disease. The central quality feature is the close interdisciplinary collaboration of pain medicine, physiotherapy and psychology, which requires a complex organizational and coordination process, especially in team meetings and final discussions.

**Objectives:**

The (different) views of the professional groups involved are brought together in the team process as a common consensus. The process of shaping the interaction of the professional groups among each other in the team meeting and final discussion as well as with the patients is examined (qualitatively) and discussed.

**Methods:**

In PAIN2020, a workshop on IMA was held to jointly reflect on the insights and experiences gained in the process so far through monitoring with staff or teams of the PAIN2020 centers. In one of three work phases, interprofessionally composed groups gathered statements from participants on the design of the interaction in team meeting and final discussion in three rotating rounds within the framework of a World Café.

**Results:**

It was possible to identify conducive and obstructive factors for the design of interdisciplinary collaboration in team meetings and final discussions, which were brought together in a superordinate framework model.

**Discussion:**

The provision of the new care service as an interdisciplinary task in a team goes beyond existing structural and process parameters in the definition of framework conditions in interdisciplinary multimodal pain therapy and should therefore also take personal competencies and professional competencies into account. Therefore, new dimensions arise for the implementation of the IMA, which should be discussed in the future.

**Graphic abstract:**

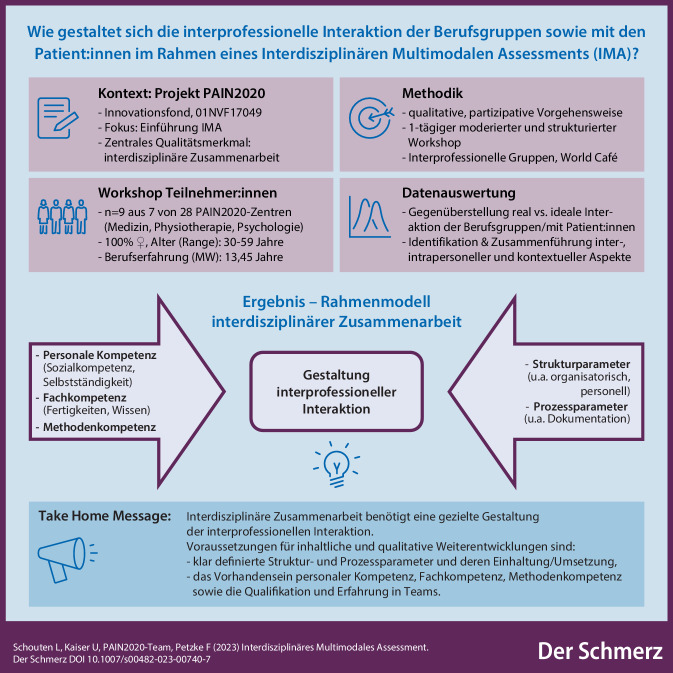

Mit dem Projekt PAIN2020 der Deutschen Schmerzgesellschaft e. V., (*P*atientenorientiert. *A*bgestuft. *I*nterdisziplinär. *N*etzwerk.; 01NVF17049, Laufzeit: 04/2018 bis 03/2022) – gefördert durch den Innovationsfonds und in Zusammenarbeit mit schmerzspezialisierten Einrichtungen der Versorgung und der BARMER – wurde eine neue Versorgungsform frühzeitig im Krankheitsverlauf bei Patient:innen mit Risikofaktoren für eine Chronifizierung eingeführt: das (ambulante) Interdisziplinäre Multimodale Assessment (IMA).

Das Ziel des Interdisiplinären Multimodalen Assessments (IMA) ist die Vermeidung einer Chronifizierung von Schmerzen im Sinne einer Sekundärprävention [8]. Im Rahmen der Konzeption der neuen Versorgungsform wurden bisher existierende Empfehlungen und Qualitätsmerkmale zu Struktur- und Prozessvoraussetzungen [1–3, 12] zur Umsetzung interdisziplinärer Diagnostik- und Therapieangebote für Patient:innen mit chronischen Schmerzen (vorrangig (teil-)stationär) an die neue Zielgruppe, Patient:innen mit nichtchronischen Schmerzen, angepasst.

Zentrale Qualitätsmerkmale des IMA sind die zeitlich und räumlich enge interdisziplinäre Zusammenarbeit sowie die gemeinsame Zielstellung der beteiligten Berufsgruppen der Schmerzmedizin [18], Physiotherapie und Psychologie/Psychotherapie [3, 8]. An eine je 60-minütige berufsgruppenspezifische Befunderhebung zur Erfassung der Beschwerden der Patient:innen schließen sich eine integrativ-interdisziplinäre Teamsitzung (20 min) und ein gemeinsames Abschlussgespräch mit den Patient:innen an (ca. 15 min) [3, 8]. „Die Qualität des IMA ergibt sich aus der Zusammenschau, der Abstimmung und der gemeinsamen Wertung der Untersuchungsergebnisse durch die beteiligten Fachdisziplinen. Die daraus resultierende gemeinsame Einordnung der individuellen Schmerzstörung ist die Basis für das weitere therapeutische Vorgehen“ [3]. Nicht selten zeigen sich im Teamprozess unterschiedliche Sichtweisen der beteiligten Berufsgruppen, wodurch ein Konsens im Team notwendig wird. Die bisherigen Ausführungen bzw. die Erfahrung im Rahmen der Projektumsetzung in PAIN2020 zeigen, dass das Erbringen der neuen Versorgungsleistung als eine gemeinsame Aufgabe im Team eines komplexen Organisations- bzw. Abstimmungsprozesses bedarf, der über bestehende Struktur- und Prozessparameter hinausgeht und interpersonelle (z. B. Kommunikation der Berufsgruppen untereinander) sowie intrapersonelle Aspekte (z. B. fachliche oder soziale Kompetenz) in der Teamzusammenarbeit voraussetzt. Aus dem Bereich der Pflege oder der Rehabilitation gibt es bspw. Hinweise darauf, dass sich Strukturen und Prozesse positiv auf die interdisziplinäre Zusammenarbeit auswirken, dass zeitgleich aber auch fachliche oder soziale Kompetenzen eine Rolle spielen [9, 10]. Für den Bereich der Schmerzmedizin gibt es hierfür aktuell keine Untersuchungen.

Im Rahmen des umfassenden Monitoringkonzeptes in PAIN2020 wurden Daten zu Struktur- und Prozessparametern im IMA erhoben [8], andererseits fanden auf Grundlage der strukturierenden Dokumentation der teilnehmenden Berufsgruppen und des Teams erste deskriptive Analysen zu Teamprozessen im IMA statt [5, 13, 14]. Im Rahmen eines Workshops zum IMA mit teilnehmenden PAIN2020-Zentren wurden u. a. die bisher gesammelten Erkenntnisse und Ergebnisse zu den Entscheidungsprozessen der Berufsgruppen und innerhalb des Teams, zu Struktur- und Prozessparametern sowie die Frage der Gestaltung von Interaktion in Teamsitzung und Abschlussgespräch besprochen. Unter Interaktion verstehen wir einen wechselseitigen Austauschprozess zwischen zwei oder mehreren Personen [16].

Mit einem Teil der Ergebnisse aus dem Workshop konnten wir bereits zeigen, dass Struktur- und Prozessparameter wichtig für eine kontinuierliche Zusammenarbeit und Interaktion im Team sind und dass Entscheidungsprozesse der Berufsgruppen im Team zusammengeführt werden [15]. Dieser Beitrag soll daher beantworten, *wie* sich die Interaktion der Berufsgruppen aus Sicht der Teilnehmenden des Workshops während der Teamsitzung und des Abschlussgesprächs sowie mit den Patient:innen im Rahmen des Abschlussgesprächs gestaltet und ob bzw. welche interpersonellen, kontextuellen und intrapersonellen Aspekte Einfluss auf die Teamzusammenarbeit haben könnten.

## Methoden

Es wurde ein qualitatives Studiendesign in Anlehnung an die Vorstufen der Partizipation (5. Stufe) des Stufenmodells zur Partizipation für Gesundheitsförderung von Wright et al. [17] ausgewählt. Durch die partizipative Herangehensweise wird eine gemeinsame Gestaltung des Untersuchungsprozesses ermöglicht, in welchem Teilnehmende als Beratende im Sinne der informierten Mitsprache fungieren [4]. Als Erhebungsmethode kam das Format eines moderierten und strukturierten Workshops zum Einsatz mit dem Ziel, Themen möglichst facettenreich und interprofessionell zu diskutieren.

Im Rahmen eines PAIN2020-Workshops zum IMA in Berlin im September 2021 wurden daher die bisher im Prozess gewonnenen Erkenntnisse und Erfahrungen des Monitorings und der strukturierenden Dokumentation in der Umsetzung der neuen Versorgungsform mit Therapierenden aus den PAIN2020-Zentren gemeinsam reflektiert und aufgekommene Fragen beantwortet. Die Berufsgruppen der Medizin, Physiotherapie und Psychologie/Psychotherapie sowie die Dokumentationsassistenzen der zum Zeitpunkt Mai–Juli 2021 aktiven 28 PAIN2020-Zentren wurden zur Teilnahme per Mail und über die individuelle Zentrenbetreuung eingeladen.

Eine detaillierte Beschreibung des Vorgehens entnehmen Sie bitte Schouten et al. [15].

### Samplebeschreibung und Datenerhebung

Für eine ausführliche methodische und inhaltliche Gesamtdarstellung des Workshops, inkl. des Zeit- und Ablaufplans, der Ziele, Fragestellungen und bisheriger Ergebnisse, verweisen wir auf Schouten et al. [15].

#### Teilnehmende

An dem Workshop zum IMA nahmen vier Ärzt:innen, drei Therapeut:innen der Psychologie/Psychotherapie und zwei Therapeut:innen der Physiotherapie aus insgesamt sieben der 28 PAIN2020-Zentren teil, die allesamt im Kontext der Interdisziplinären Multimodalen Schmerztherapie (IMST) tätig sind. Es handelt sich dabei um drei Einrichtungen der Maximalversorgung, drei Einrichtungen aus der Normalversorgung sowie eine ambulante Einrichtung aus insgesamt sechs Bundesländern (Bayern, Hessen, Niedersachsen, Nordrhein-Westfalen, Rheinland-Pfalz und Sachsen).

Fünf der Teilnehmenden waren zwischen 30 und 39 Jahren, zwei zwischen 40–49 sowie zwei zwischen 50 und 59 Jahren alt. Die durchschnittliche Berufserfahrung betrug 13,45 Jahre (Minimum: 3 Jahre, Maximum: 30 Jahre). Acht der insgesamt neun Teilnehmenden hatten bereits eine schmerzspezifische Weiterbildung (Spezielle Schmerztherapie, Spezielle Schmerzpsychotherapie oder Spezielle Schmerzphysiotherapie) absolviert.

In den Einrichtungen, die am Workshop teilnahmen, wurden im Zeitraum von April 2019 bis Juli 2021 zwischen 7 und 60 IMAs umgesetzt. Von diesen haben die Teilnehmenden im Durchschnitt ca. 12 IMAs durchgeführt (*n* = 7; Minimum: 5 IMAs, Maximum: 30 IMAs).

#### Moderierende

Die Moderation des Workshops erfolgte durch drei Mitglieder des Projektteams (die Autor:innen FP, LS, UK), die an der Konzeption der neuen Versorgungsleistung beteiligt waren und ergänzend eine schmerzspezifische Zusatzqualifikation, Moderations- sowie klinische Erfahrung in der Durchführung der Assessments mitbringen.

#### Vorbereitung des Workshops

Der Workshop wurde in Zusammenarbeit mit dem Projektteam PAIN2020 strukturell, inhaltlich und methodisch konzipiert. Als Ergebnis des Austauschprozesses entstanden zwei Handbücher: Mit dem Handbuch für die *Teilnehmenden* wurden im Vorfeld die organisatorischen Rahmenbedingungen, die Zielstellung sowie die Inhalte des Workshops und bisherige Auswertungen zum Teamprozess im IMA [5, 13, 14] zur Verfügung gestellt. Das Handbuch für die *Moderierenden* beinhaltete ergänzende Informationen zum Verhalten und den Aufgaben der Moderation während des Workshops, zur geplanten Dokumentation der jeweiligen Arbeitsphasen (u. a. Fotodokumentation, schriftliche Notizen), eine Beschreibung zu den ausgewählten Methoden sowie die expliziten Ziel- und Fragestellungen der jeweiligen Arbeitsphasen.

### Datenerhebung – Ablauf des Workshops

Der Workshop unterteilte sich nach einem *Einstieg zum Ankommen und Kennenlernen* in *drei Arbeitsphasen*:

- *Arbeitsphase 1****:*** *Vor- und Nachteile der professionsspezifischen „Instrumente“ zum Einschluss und zur Befundung im IMA*
- *Arbeitsphase 2****:*** *Handhabung der Entscheidungskriterien (berufsgruppenspezifisch und -übergreifend)*
- *Arbeitsphase 3: Gestaltung der Teamsitzung und des gemeinsamen Abschlussgesprächs, Entscheidungsprozesse im Team (interdisziplinär/‑professionell)*

**Figure 1:**
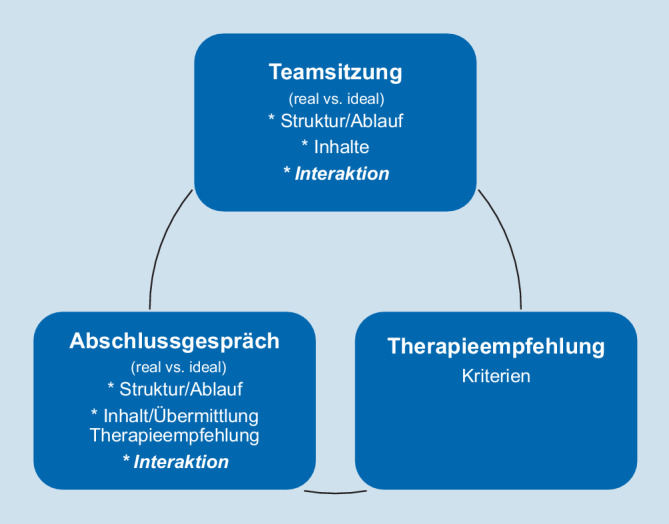

Inhalte der Arbeitsphase 3 waren der Austausch über Strukturen/Abläufe, Inhalte und die Gestaltung der Interaktion in Teamsitzung und Abschlussgespräch (aus realer und idealer Perspektive) sowie die Erfassung von Kriterien zur Formulierung einer gemeinsamen Therapieempfehlung und die dazugehörigen Entscheidungsprozesse im Team (vgl. Abb. 1).

In interprofessionell zusammengestellten Gruppen wurden zu den im Vorfeld definierten Fragestellungen in drei rotierenden Durchgängen (World Café) die Aussagen der Teilnehmenden auf Karteikarten bzw. direkt auf der Moderationswand zusammengetragen. Nach jeder Rotation wurden einleitend die Ergebnisse der jeweils vorherigen Gruppe(n) zusammengefasst und anschließend durch die weiteren Teilnehmenden ergänzt, bekräftigt oder hinterfragt (keine Zustimmung zu dem Ergebnis). Die einzelnen Arbeitsschritte wurden durch die Moderierenden mit (Zwischen‑)Fotos und eigenen Notizen dokumentiert. Ergänzend liefen in jeder Arbeitsphase Audioaufnahmen mit, um im Nachgang noch offene Punkte auf den Karteikarten der Fotodokumentation zu rekonstruieren.

Dieser Artikel beschreibt ausgewählte Ergebnisse der Arbeitsphase 3 mit den Fragestellungen:

- Wie gestaltet sich die Interaktion der Berufsgruppen während einer Teamsitzung? *(real vs. ideal)*
- Wie gestaltet sich die Interaktion mit den Patient:innen und den Berufsgruppen? *(real vs. ideal)*

### Datenauswertung

Die Datenauswertung gliederte sich in zwei übergeordnete Teile. Im ersten Teil wurden die Ergebnisse der Fotodokumentation und der Notizen zur Gestaltung der Interaktion der Berufsgruppen durch die Moderierenden in eine Excel-Tabelle überführt. Im Anschluss daran folgte eine Verschriftlichung der Ergebnisse anhand der bereits vorformulierten Fragestellungen und eine Aufbereitung der inhaltlichen Schwerpunkte der Diskussionen.

Im Rahmen des zweiten Auswertungsteils wurde ein vorläufiges Rahmenmodell entwickelt, das dazu dienen soll, erste Perspektiven, strukturelle und prozessuale Abläufe oder Komponenten der interprofessionellen Interaktion im IMA miteinander zu verknüpfen. Dies wurde für jeden Auswertungsschritt unabhängig durch zwei Autorinnen (LS, UK) durchgeführt, bei Uneinigkeit sollte ein Dritter (FP) hinzugezogen werden.

Nachdem die von den Teilnehmenden genannten Aspekte von den Karteikarten in einem ersten Schritt durch die Autorinnen getrennt voneinander zu ersten Bereichen zugeordnet werden konnten, wurden die Ergebnisse durch die Autorinnen in Kategorien zusammengeführt. Aus der Zusammenführung ergaben sich erste Ideen für Überkategorien, deren Übertragbarkeit auf bisherige Konzeptarbeiten (Identifikation über Recherche) in einem nächsten Schritt geprüft wurde. Daran anschließend erfolgte die Festlegung von Überkategorien, die in identifizierte Konzeptarbeiten zu den Kompetenzbereichen des Deutschen Qualifikationsrahmens [6], den Kontextfaktoren [7] sowie den Struktur- und Prozessparametern der Interdisziplinären Multimodalen Schmerztherapie [11, 12] eingeordnet werden konnten (vgl. Tab. 1). Die Aussagen der Teilnehmenden bzw. die entstandenen Kategorien wurden erneut in einem getrennten Verfahren durch die Autorinnen den einzelnen Überkategorien zugeordnet und abschließend die Zuordnung diskutiert und finalisiert. In einem vierten Schritt wurden die Überkategorien in einem Rahmenmodell zusammengeführt, das die unterschiedlichen Perspektiven der Teilnehmenden im Hinblick auf die Interaktion in Teamsitzung und Abschlussgespräch zusammenführt. Ergänzend wurden die Relationen der Überkategorien dargestellt, die sich aus der Überlappung der Zuordnung von einzelnen Aussagen der Teilnehmenden zu den Überkategorien ergaben.

**Table 1:** 

Kompetenzbereich	Beschreibung (inkl. Beispiele aus Arbeitsphase 3)
Fachkompetenz [6]	„*Fachkompetenz* beschreibt die Fähigkeit und Bereitschaft, Aufgaben und Problemstellungen eigenständig, fachlich angemessen methodengeleitet zu bearbeiten und das Ergebnis zu beurteilen.“ Sie wird unterteilt in: „*Wissen*“: Gesamtheit der Fakten, Grundsätze, Theorien und Praxis in einem Lern- oder Arbeitsbereich als Ergebnis von Lernen und Verstehen. Wissen beinhaltet auch fachspezifisches und/oder fachtheoretisches Wissen, das bei der Bewältigung von Aufgaben hilft und dessen Erwerb ein lebenslanger Prozess ist. Wissen kann auch in Tiefe und Breite unterteilt werden. „*Fertigkeiten*“: Fähigkeit, Wissen anzuwenden und Know-how einzusetzen, um Aufgaben auszuführen und Probleme zu lösen. Sie können als kognitive oder praktische Fähigkeiten beschrieben werden. Eine Unterteilung in instrumentale Fertigkeiten, systemische Fertigkeiten und Beurteilungsfähigkeit ist möglich
Personale Kompetenz [6]	„*Personale Kompetenz* bezeichnet die Fähigkeit und Bereitschaft, sich weiterzuentwickeln und das eigene Leben eigenständig und verantwortlich im jeweiligen sozialen, kulturellen bzw. beruflichen Kontext zu gestalten.“ Sie wird unterteilt in: „*Sozialkompetenz***“:** zielorientierte Zusammenarbeit, Erfassung von Interessen und sozialen Situationen inkl. rationaler und verantwortungsbewusster Auseinandersetzung und Verständigung, Mitgestaltung der Arbeits- und Lebenswelt. Zur Sozialkompetenz gehören Team‑/Führungsfähigkeit und Kommunikation. „*Selbstständigkeit*“: Eigenständiges und verantwortliches Handeln, Reflexion des eigenes Handeln und des Handelns der anderen, Entwicklung der eigenen Handlungsfähigkeit. Zur Selbstständigkeit gehören Eigenständigkeit, Verantwortung, Reflexivität und Lernkompetenz
Methodenkompetenz [6]	Fähigkeit, an Regeln orientiert zu handeln. Dazu gehört auch die reflektierte Auswahl und Entwicklung von Methoden. Das heißt u. a. auch, Arbeitstechniken, Verfahrensweisen und Lernstrategien sachgerecht, situationsbezogen und zielgerichtet gebrauchen zu können. „Fachkompetenz“ und „personale Kompetenz“ schließen Methodenkompetenz mit ein (im Sinne von Querschnittskompetenz)
Kontextfaktoren [7]	Kontextfaktoren sind Faktoren, die auf einen bestimmten Bereich sowohl positiven als auch negativen Einfluss haben können. Der Begriff Kontextfaktor kommt ursprünglich aus der ICF-Denkweise und beschreibt die Einflussfaktoren einer betreffenden Person, die positiven (Förderfaktoren) oder negativen (Barrieren) Einfluss auf die Auswirkung der Teilhabe an bestimmten Lebensbereichen haben
Strukturparameter IMST [11, 12]	Parameter zur Beschreibung von Rahmenbedingungen, wie: organisatorische, materielle und personelle Ressourcen. *organisatorisch*: u. a. Art der Umsetzung *personell*: u. a. Verfügbarkeit von Therapeut:innen oder Qualifikation(en)
Prozessparameter IMST [11, 12]	Parameter zur Beschreibung der Umsetzung und der Abläufe, das heißt, „wie“ Leistungen erbracht werden. Hierzu gehören u. a. die Dokumentation, die Gestaltung der interdisziplinären Zusammenarbeit, das evidenzbasierte Vorgehen und die Teilnahme an externer Qualitätssicherung. *Dokumentation*: Art, Standardisierung, Zeitpunkte *Gestaltung der interdisziplinären Zusammenarbeit*: z. B. diagnostisches Assessment, Teambesprechung *Evidenzbasiertes Vorgehen*: z. B. Leitlinien

## Ergebnisse

In der Ergebnisdarstellung werden beispielhafte Aussagen der Teilnehmenden (/*Inhalt Karteikarte*/, [*Ergänzung zum Verständnis der Karteikarte*] und „*wörtliches Zitat einer:s Teilnehmenden*“) aufgeführt, um die Nachvollziehbarkeit der Ergebnisse zu erleichtern.

### Interaktion Teamsitzung

Die Gestaltung der Interaktion in der Teamsitzung wurde durch die Teilnehmenden *nicht* direkt in *real* und *ideal* unterschieden, da dies in den unterschiedlichen Einrichtungen variiert und nicht „verallgemeinert“ werden kann – daher erfolgte durch die Teilnehmer:innen eine Unterteilung in *förderliche* und *hinderliche* Faktoren.

Die Teilnehmenden benannten als *förderliche Faktoren für die Interaktion in der Teamsitzung*:

- /den anderen Fragen stellen/ (u. a. zur Wahrnehmung der Patient:innen)
- /offenes Zuhören + Interesse für die Perspektiven der Anderen/ (v. a. Respekt vor der Kompetenz der Anderen)

Die Teilnehmenden wünschen sich noch mehr /Offenheit für fachübergreifende Interaktion/ und je nach Teamzusammensetzung einen /respektvoller[en] Umgang/, eine /wertfreie/ Interaktion, /Wertschätzung/ sowie einen Raum, eigene Gedanken zu äußern, die auch von den Gedanken/Meinungen der anderen Berufsgruppen abweichen (/Raum bekommen Gedanken zu äußern/).

Die Teilnehmenden benannten als *hinderliche Faktoren für die Interaktion in der Teamsitzung*:

- /„Gefahr“ sich in Details zu verlieren/ bzw. /im Kreis drehen/
- z. T. (in Bezug auf die zur Verfügung stehende Zeit) fehlender /Raum für gegenseitige Anregungen/ (fachübergreifend), was wiederum mit Schwierigkeiten bei der Findung der Therapieempfehlung einhergeht
- /teilweise spürbarer Zeitdruck/ (d. h., zeitliche Limitierung führt dazu, dass das Gespräch „verbal“ limitiert wird – „müssen jetzt mal zum Punkt kommen“ – bzw. das Gespräch unterbrochen werden muss, da noch offene Punkte wie z. B. Dokumentation anstehen)

Die Teilnehmenden beschreiben einen interaktionell /„spannende[n] Punkt“, wenn es zu Widersprüchen bzw. unterschiedlichen Meinungen im Team kommt/, die u. a. mit der /Bereitschaft [einhergehen] „sich umzuentscheiden“/. Das Um-Entscheiden geschieht u. a. aufgrund von mehr Informationen durch die Teamsitzung, durch patientenbezogene Faktoren, schlüssige Schlussfolgerungen bzw. das Einlassen auf die Meinung der anderen Berufsgruppen.

Bei *Nicht-Einigung* werden unterschiedliche Wahrnehmungen zu den Patient:innen mit in das Abschlussgespräch genommen. Hier wünschen sich die Teilnehmenden, dass die eigenen Grenzen bzw. die Grenzen der anderen Berufsgruppen noch mehr respektiert werden (/eigene Grenzen respektieren/Grenzen der Berufsgruppen respektieren/).

Ergänzend wurden durch die Teilnehmenden übergeordnete Aspekte aufgeführt, die *Einfluss auf den Teamprozess* haben (Zustimmung zu den Punkten über alle drei Kleingruppen hinweg):

- /Routine/ (d. h. Interaktion ist „abhängig davon, wie das Team bereits zusammenarbeitet bzw. wie die Struktur oder der Ablauf in einer Einrichtung bereits etabliert ist“)
- /Zeitfaktor/ (i. S. v. spürbarem Zeitdruck, Relevanz für eigene Themen wird ggf. nicht gesehen durch die anderen Berufsgruppen, kein Raum für Unsicherheit in der eigenen Einschätzung)
- /Personen/ (Charakter/Persönlichkeiten)
- /Häufigkeit der Zusammenarbeit/ (s. Routine, bei Personen nochmals ergänzt)
- /Kennen der „Besonderheiten“ der Personen/ (*Eigenschaften*)
- /Stellenwert, der *einer Person* zugeschrieben wird/
- /„neue“ Person/
- /Hierarchie/ (in Bezug auf die Stellung der eigenen Person, ihre Erfahrung und Kompetenz und u. a. auch die Kultur/der Umgang in der Einrichtung)

Aus den Rückmeldungen der Teilnehmenden ergab sich zusammengefasst in Bezug auf die Teamsitzung, dass vor allem grundlegende Gesprächsführungskompetenzen und -abläufe wichtig sind, insbesondere in der Moderation und Führung der Teamsitzung. Rollenvorstellungen und -erwartungen (direkt, indirekt) wird eine wesentliche Rolle in der Interaktion zugeschrieben.

### Interaktion – Abschlussgespräch

In der Erarbeitung der Interaktion im Spannungsfeld zwischen *real* und *ideal* kristallisierten sich über die drei Durchgänge übergeordnete Inhalte im Rahmen des Abschlussgesprächs heraus. Wesentliche Kernaspekte im *realen* Zustand waren dabei:

- Die Herausforderung der Teamgesprächsführung (im Vergleich zur Einzelgesprächsführung)
- Die Rollenverständnisse und Hierarchien in der professionellen Interaktion
- Das Rollenverständnis der Betroffenen als Patient:in

Bezüglich der *Herausforderung der Teamgesprächsführung* zeigt sich in der Beschreibung der realen Interaktion ein bisher wenig integratives Vorgehen (bspw. wenn immer der:die Arzt:Ärztin beginnt oder jede:r für sich spricht). Dies leitet direkt auf die Ebene der *Hierarchien und Rollenverständnisse* über, in denen der:die Arzt:Ärztin als dominant beschrieben wurde, allerdings von ärztlicher Seite auch berichtet wurde, dass „die anderen Professionen sich hinter mir verstecken“ bzw. eher zurückhaltend in der Gesprächsführung sind. Problematisch war auch hier, dass häufig Professionen sowohl in der Teamsitzung als auch im Abschlussgespräch fehlten.

Demgegenüber wurden *Betroffene in ihrer Rolle als Patient:in* als offen beschrieben. Sie hätten sich ernst genommen gefühlt, manchmal aber auch festgesetzte Vorstellungen gehabt. Eine Einrichtung begrüßte die Patient:innen während des Abschlussgespräches im Kreis, um den Gedanken der Integration zu betonen.

Für die *ideale* Interaktion wurde mit /Zeit schaffen/ die Notwendigkeit ausreichender Zeit sowohl auf die Durchführung des Gesprächs (im Sinne der Motivationsförderung) als auch auf die Nachbesprechung im Team und Möglichkeit der Rückmeldung bezogen. Insbesondere in der Anfangsphase des Projekts wurde Zeit für die Nachbesprechung, Abstimmung und Rückmeldung als hilfreich erlebt, um Interaktionsabläufe zu reflektieren und eine konstruktive Dynamik innerhalb des Teams zu fördern.

Der *ideale* /interaktive Umgang im Team/ wurde verstanden als /flexible Gesprächsführung unter den Professionen/ sowie die Berücksichtigung/Anwendung des Konzeptes des *shared decision making*.

Es wurde aber in den beiden ersten Kernaspekten sichtbar, dass es für die Entwicklung eines gelungenen Abschlussgesprächs neben Zeit auch eine /konstruktive Feedbackkultur/ im Team braucht, die ihrerseits Zeit für Übung, Erfahrung und die Entwicklung von Sicherheit in sich als Mitglied und in Bezug auf die anderen Mitarbeitenden erfordert.

Aufbauend auf die Gedanken zum *shared decision making* wurde die *ideale* Interaktion mit Betroffenen als Patient:in auf Augenhöhe verstanden – als Gleichberechtigte:n im Kreis, dem/der mit Wertschätzung begegnet wird (/Interaktiver Umgang mit Patient/).

Zum *idealen* /interaktive[n] Gesprächsablauf/ wurden verschiedene Strategien gezählt, die idealerweise immer angewendet werden. Dazu gehörten eine kurze Einleitung zum Abholen der Patient:innen, das Erfragen der Perspektive des/der Patienten:in in Hinblick auf sein:ihr Erleben und Schmerzmodell, die Einleitung zur Vorstellung des gemeinsamen (teambasierten) Schmerzmodells sowie eine Überleitung bzw. Verbindung der Professionen im Gesprächsverlauf.

Zusammengefasst kamen in dieser Diskussion vor allem Reflexionen zu Rollenverständnis und Hierarchien auf, die zwar gewachsen und gewohnt waren, aber in der Durchführung interdisziplinärer, integrativer Abschlussgespräche oft als hinderlich erlebt wurden. Darüber hinaus wurde auch hier deutlich, dass Gesprächsführungskompetenzen für alle Teammitglieder sowie die Klärung und bewusste Planung der Moderation des Abschlussgesprächs wünschenswert sind. Dabei spielen auch die Erfahrungen der Betroffenen als Patient:innen im Gesundheitswesen eine große Rolle, die nicht selten andere Dinge erwarten, als im idealen Gesprächsverlauf (bspw. Gesprächspartner:innen auf Augenhöhe, Eigenverantwortung in der Entscheidungsfindung und -annahme) gewünscht wurden.

### Übermittlung der Therapieempfehlung – Abschlussgespräch

Erfahrungen aus der *realen* Umsetzung in Bezug auf die Interaktion zur Übermittlung von Therapieempfehlungen lassen sich in drei Kernbereiche gliedern (mit Überschneidungen zur Interaktion im Abschlussgespräch):

- Voraussetzungen für die Entscheidungsfindung mit Patient:innen
- Die Struktur bzw. der Ablauf der interprofessionellen Informationsvermittlung
- Die Rolle (des:der Einzelnen des Teams) bei der Vermittlung der Therapieempfehlungen

Einige Teilnehmende berichteten, dass sie als Team „laut vor den Betroffenen dachten“, um so Begründungen, Abwägungen oder Gedankengänge für diese sicht- und nachvollziehbar zu gestalten. Sie berichteten, dass dies die Entscheidungsfindung des:der Patient:in unterstützte. Wiederholt wurde thematisiert, dass Unsicherheiten darüber bestehen, „wer am Ende die Entscheidung trifft“ bzw. wie damit umgegangen werden kann, wenn im Abschlussgespräch keine abschließende Entscheidung getroffen werden konnte. Dieser Zustand wurde als sehr unbefriedigend erlebt.

Als Herausforderung wurde beschrieben, ein angemessenes Maß an interprofessionellen Informationen zu finden, das ausreichend Basis für ein umfassendes Verständnis und die Entscheidung für die Patient:innen liefert, gleichzeitig aber nicht durch die damit einhergehende Komplexität überfordert. In diesem Zusammenhang wurde sich erneut Zeit in der Vorbereitung auf das Abschlussgespräch gewünscht, um eine gemeinsame interprofessionelle Abwägung und Priorisierung vornehmen zu können.

Nicht zuletzt war das Rollenverständnis der Teammitglieder auch hier von Bedeutung, vor allem in Bezug auf die Führung des Gesprächs, aber auch in Bezug auf die Übermittlung guter oder schlechter Nachrichten.

Im *idealen* Bereich wurde erneut die Bedeutung einer (professionellen, ausgewogenen) Gesprächsführung benannt. Dabei wurde der Wunsch formuliert, mit den Patient:innen interaktiv und transparent den Entscheidungsprozess zu gestalten. Andere Aspekte in diesem Zusammenhang betrafen aber tatsächlich auch den Umgang im Miteinander innerhalb des Teams. Von Bedeutung erschienen bspw. die Offenheit der Professionen untereinander für fachübergreifende Ergänzungen (bspw. wenn der:die Psychologe:in/Psychotherapeut:in Aspekte der *fear avoidance* in Ergänzung zur physiotherapeutischen Erklärung liefert). Hier spielten auch die Ergebnisoffenheit des Abschlussgesprächs selbst sowie die Offenheit und Transparenz den Patient:innen gegenüber hinein (bspw. direkt nach anderen Ansichten auf dessen/deren Seite zu fragen). Eine Moderation für Entscheidungsprozesse wurde als notwendig angesehen – für die es Erfahrung und möglicherweise Gesprächsführungskompetenz braucht.

Die Ergebnisse hinsichtlich der Interaktion mit den Patient:innen bzw. der Übermittlung der Therapieempfehlung zeigen (ergänzend zu den Aspekten aus Teamsitzung und Abschlussgespräch), dass es notwendig erscheint, Zeit in der Vorbereitung einzuplanen, um entsprechende Prozesse vorzustrukturieren, Informationen und Empfehlungen zu priorisieren und nächste Schritte im Team festzulegen (z. B. wer sagt im Team was wann zu wem). Diese sollten möglichst auf die bekannten Befunde der Patient:innen aufbauen. Es wurden auch schriftliche Empfehlungen direkt für die Patient:innen angedacht, die als Gedankenstütze dienen könnten.

### Integration der Ergebnisse in ein Rahmenmodell

**Figure 2:**
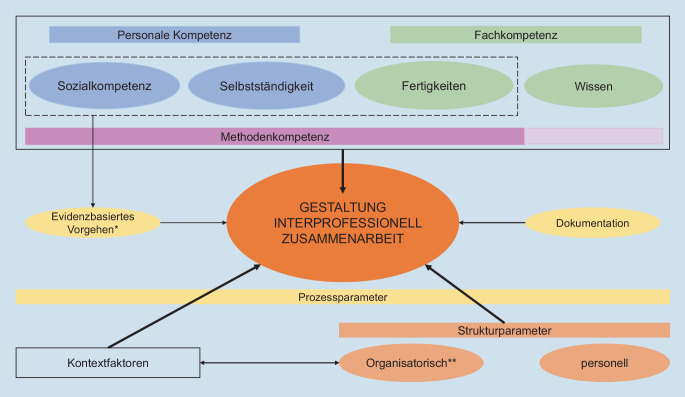

Im zweiten Schritt der Datenauswertung wurde durch zwei der Autor:innen (LS, UK) eine erste beispielhafte Idee eines Rahmenmodells entwickelt, das die zuvor beschriebenen Ergebnisse zur Interaktion aus Teamsitzung und Abschlussgespräch zusammenfasst und den Einfluss auf die Gestaltung interdisziplinärer Zusammenarbeit beschreibt (vgl. Abb. 2). Ergänzend wurden die Beziehungen der übergeordneten Kategorien auf die Gestaltung interdisziplinärer Zusammenarbeit im IMA dargestellt. Relationen zwischen einzelnen Überkategorien wurden dann kenntlich gemacht, wenn mindestens drei überlappende Aussagen der Teilnehmenden den Überkategorien zugeordnet werden konnten. Die Stärke der Pfeile in der Abbildung ergibt sich aus der Häufigkeit der Zuordnung/Nennung der Aussagen der Teilnehmenden zu den Überkategorien, die in Tab. 2 dargestellt werden. Ergänzend werden Beispiele für die Überkategorien angefügt. Die Beschreibung der Überkategorien finden sich in Tab. 1.

**Table 2:** 

Unterteilung in (Überkategorien)		Häufigkeit der Zuordnung (*n*)	Beispiele
*Fachkompetenz*	Wissen	10	/Erfahrung/, /Routine/
	Fertigkeiten	*15*	/flexible Gesprächsführung/, /grundlegende Gesprächsführungskompetenzen und -abläufe/, /Offenheit für fachübergreifende Ergänzungen/
*Personale Kompetenz*	Sozialkompetenz	*17*	/eigene Grenzen bzw. Grenzen der anderen respektieren/, /wertschätzende Grundhaltung/, /Offenheit für fachübergreifende Interaktion/
	Selbstständigkeit	*19*	/Feedbackkultur (innerhalb des Teams)/, /Häufigkeit der Zusammenarbeit/, /Rollenvorstellungen und -erwartungen/
*Methodenkompetenz*		10	/flexible Gesprächsführung/, /grundlegende Gesprächsführungskompetenzen und -abläufe/
*Kontextfaktoren*		7	/spürbarer Zeitdruck/, /geringe Zeit/keine zeitlichen Ressourcen/
*Strukturparameter*	Organisatorisch	*12*^b^	/inhaltliche Vor- und Nachbereitungszeit/, /Termin- und Pünktlichkeitsmanagement der beteiligten Berufsgruppen/, /keine zeitlichen Ressourcen/, /mangelnde Konstanz der Teilnahme von Seiten der Berufsgruppen/
	Personell	*12*	
*Prozessparameter*	Dokumentation	5	/geringe Zeit für Dokumentation/
	Evidenzbasiertes Vorgehen	5^a^	/Feedbackkultur/

^a^ Kriterien aus dem Abschlussgespräch (100 %)

^b^ Überwiegend (>/= 66 %) Kriterien aus der Teamsitzung

Kursiv hervorgehoben**:** die fünf häufigsten genannten Kategorien

Im Fokus der Teilnehmenden während der Diskussion stand vor allem die Frage der *Gestaltung interdisziplinärer Zusammenarbeit*, die den Mittelpunkt des Rahmenmodells bildet (vgl. Abb. 2). Die Gestaltung der interdisziplinären Zusammenarbeit wird durch Struktur- und Prozessparameter sowie begleitende Kontextfaktoren beeinflusst, die sich im unteren/mittleren Teil der Abbildung befinden. Die von den Teilnehmenden beschriebenen *Strukturparameter* konnten *organisatorischen* (z. B. /inhaltliche Vor- und Nachbereitungszeit/) und *personellen* (z. B. /mangelnde Konstanz der Teilnahme von Seiten der Berufsgruppen/) Aspekten zugeordnet werden. Insbesondere organisatorische Strukturparameter scheinen in der Teamsitzung gegenüber dem Abschlussgespräch zunächst von großer Bedeutung zu sein. Ergänzend konnten begleitende *Kontextfaktoren* identifiziert werden, die wiederrum mit den organisatorischen Strukturparametern in Verbindung stehen. Zu diesen zählt bspw. /spürbarer Zeitdruck/. Die von den Teilnehmenden beschriebenen *Prozessparameter* konnten dem Bereich der *Dokumentation* (z. B. /geringe Zeit für Dokumentation/) und dem *Evidenzbasierten Vorgehen* zugeordnet werden, wobei Letzteres nur aus dem Abschlussgespräch zugeordnet werden konnte.

Ergänzend kristallisierte sich aus den Ergebnissen zur Interaktion in Teamsitzung und Abschlussgespräch heraus, dass personale Kompetenzen, Fachkompetenzen bzw. eine Methodenkompetenz [6] Einfluss haben auf die Gestaltung der interdisziplinären Zusammenarbeit (vgl. Abb. 2). Da die Aussagen der Teilnehmenden mehrfach zu den verschiedenen Kompetenzbereichen zugeordnet werden konnten, sind diese in der Abbildung mit einem Kasten umrandet. Die Aussagen der Teilnehmenden konnten am häufigsten der Sozialkompetenz, der Selbstständigkeit und den Fertigkeiten zugeordnet werden, weshalb diese nochmals gesondert mit einem Kasten umrahmt sind.

Die *personale Kompetenz* umfasst die *Sozialkompetenz* und *Selbstständigkeit* und beinhaltet Aussagen der Teilnehmenden zu /Offenheit für fachübergreifende Interaktion/ bzw. /wertschätzende Grundhaltung/. Zur *Fachkompetenz* zählen *Fertigkeiten*, wie bspw. /flexible Gesprächsführung/ oder /grundlegende Gesprächsführungskompetenzen/, und das *Wissen*. Beim Wissen lassen sich bspw. auch Aspekte zur /Erfahrung/ mit einordnen. Die *Methodenkompetenz* umfasst – ähnlich der personalen Kompetenz und Fachkompetenz – Aspekte zur Gesprächsführung bzw. zu Gesprächsführungskompetenzen, die als nützlich für die Gestaltung interdisziplinärer Zusammenarbeit gesehen werden.

Insbesondere die personale Kompetenz, die Fachkompetenz und die Strukturparameter scheinen – in Bezug auf das teilnehmende Sample – einen Beitrag zur Gestaltung der interdisziplinären Zusammenarbeit aufzuweisen und wurden durch die Teilnehmenden hervorgehoben.

## Diskussion

Im Rahmen der hier dargestellten, ausgewählten Ergebnisse zur Gestaltung der Interaktion in Teamsitzung und Abschlussgespräch und der sich daraus ableitenden Schritte für die interdisziplinäre Zusammenarbeit aus dem Workshop zum IMA wurden Therapierende der drei beteiligten Berufsgruppen (Schmerzmedizin, Physiotherapie und Psychologie/Psychotherapie) befragt, die an der Umsetzung des IMAs im Rahmen von PAIN2020 aktiv beteiligt waren.

### Die Perspektive der Teilnehmenden

Sowohl die Teamsitzung als auch das Abschlussgespräch – hier insbesondere die Übermittlung der professionsspezifischen und der interprofessionellen Empfehlungen – zeigen sehr unterschiedlich komplex ablaufende Teamprozesse (u. a. wann werden welche Ergebnisse eingebracht, Wichtung der eigenen Profession, Informationsweitergabe und Einordnung der eigenen und anderen Befunde). Die Ergebnisse zeigen auch, dass strukturelle und prozessuale Aspekte die Gestaltung interdisziplinärer Zusammenarbeit begleiten und Einfluss auf die Interaktion der Berufsgruppen haben (u. a. aufgrund einer Erkrankung von Mitarbeitenden, Vertretung, Personalkonstanz, neue Mitarbeitende). Ergänzend werden durch die Teilnehmenden Aspekte wie wertschätzender und offener Umgang bzw. fachlicher Austausch im Team oder Gesprächsführungskompetenz benannt, die als förderlich für die Gestaltung interdisziplinärer Zusammenarbeit gesehen werden und sich den Bereichen der personalen Kompetenz und Fachkompetenz zuordnen lassen. Gleichzeitig benennen die Teilnehmenden auch hinderliche Faktoren für die Gestaltung interdisziplinärer Zusammenarbeit, die sich ebenfalls auf die zuvor beschriebenen Aspekte beziehen (z. B. fehlender fachübergreifender Austausch).

Auch wenn diese Beobachtungen und Berichte nicht im Detail vertieft werden konnten, wurde deutlich, dass Teamaspekte nur im Team geklärt werden können und die Anzahl, die Erfahrung und das Rollenverständnis der Therapierenden die Zusammenarbeit, neben den Struktur- und Prozessparametern, beeinflusst. Interessanterweise wurden interaktionell relevante Aspekte primär in Bezug auf die Teamzusammenarbeit, weniger auf die Arbeit mit den Patient:innen berichtet bzw. gewünscht.

### Erkenntnisse aus der Literatur

In der Literatur werden Determinanten erfolgreicher interprofessioneller Teamarbeit in anderen Settings beschrieben, die auch bereits u. a. in der Rehabilitation als Qualitätsmerkmale gelten. Im Rahmen einer systematischen Literaturrecherche durch Müller et al. [10] konnten mit einer Evidenz-Matrix 16 Determinanten erfolgreicher interprofessioneller Teamarbeit identifiziert werden. Davon beziehen sich sechs Determinanten auf organisationale (u. a. Vorhandensein ausreichender und qualifizierter Mitarbeiter, ausgewogene Teamzusammensetzung) und 10 auf prozessuale Aspekte (u. a. ausreichender Informationsfluss und offene Kommunikation, adäquate Koordination der Behandlungsleistung, Mitarbeiterpartizipation und Einfluss auf Entscheidungsfindung im interprofessionellen Team, angemessene Teamführung und gutes Teamklima). Die Autor:innen führen an, dass es darüber hinaus Determinanten gibt, die über die Organisation, die Führungsebene oder ausgehend vom einzelnen Teammitglied Einfluss auf die Teamzusammenarbeit ausüben. Hierzu zählen u. a. Teamkohäsion, Verhaltensnormen, Einstellung und Haltung zur Teamarbeit, Bestimmung von Teamzielen sowie Aufgaben des Rehabilitationsteams oder interprofessionelles Lernen im Team, die bisher noch nicht zugeordnet sind.

In einer Studie von Körner et al. [9], die für das Setting der Pflege von chronischen Schmerzpatient:innen eine systematische Literaturrecherche durchführten, konnten relevante Merkmale der Teamarbeit und Interventionen zur Verbesserung der interprofessionellen Teamarbeit identifiziert werden. In den 23 eingeschlossenen Studien waren die am häufigsten genannten Merkmale u. a. die Charakteristika der Teammitglieder, gemeinsame Aufgabe, Kommunikation, Kooperation, Koordination, Verantwortung, Beteiligung oder Mitarbeiterzufriedenheit.

Beide Arbeiten berichten, dass erfolgreiche Teamarbeit einen positiven Einfluss auf Patient:innen‑, Mitarbeiter:innen- und organisationsbezogene Ergebniskriterien (z. B. Behandlungsoutcome oder Mitarbeiterzufriedenheit) haben kann [9, 10]. Die Autor:innen gruppierten die benannten Merkmale zur Verbesserung der interprofessionellen Zusammenarbeit nach dem Input-Prozess-Output Modell, um die Hauptmerkmale interprofessioneller Teamarbeit zusammenzutragen. Dabei bezieht sich der Input u. a. auf die Charakteristiken der Teammitglieder, Teamzusammensetzung, Hierarche sowie ergänzend strukturelle Parameter, der Prozess u. a. auf die Kommunikation, Koordination, Konfliktmanagement sowie ergänzende prozessuale Parameter, die wiederum Einfluss auf den Teamoutput haben (u. a. Arbeitszufriedenheit, Behandlungsergebnis). Die Ergebnisse können dazu genutzt werden, die Implementation und Evaluation interprofessioneller Teamarbeit zu begleiten.

Es wäre daher zukünftig vorstellbar, auch für das IMA solch ein Input-Prozess-Output-Modell zu entwickeln und auszuformulieren, das die interprofessionelle Zusammenarbeit im Team beschreibt. In den Ergebnissen dieses Artikels lassen sich bereits erste Hinweise (übergeordnet) auf die Zuordnung und eine erste inhaltliche Ausführung zur Input- und Prozessebene des Modells finden, wie bspw. beim *Input* die Charakteristik des Teams oder die Führung, beim *Prozess *die Kommunikation oder das Rollenverständnis.

### Herausforderungen und Entwicklungspotenziale

Bisher existieren für die interdisziplinäre multimodale Schmerztherapie vor allem Empfehlungen zu Struktur- und Prozessvoraussetzungen [12] sowie zu Zielsetzungen. Dabei handelt es sich hauptsächlich um Empfehlungen für die Therapie chronischer Schmerzen, die in Deutschland vorrangig im stationären bzw. teilstationären Kontext umgesetzt werden. Auch hier sind die Herausforderungen zur Koordination der Abläufe und das Vorhalten ausreichender Strukturen (bis hin zu personeller Konstanz) enorm. Für den ambulanten Bereich gibt es erste konzeptionelle Vorüberlegungen, die bisher als Versorgungsstruktur jedoch nicht flächendeckend in Deutschland (vereinzelt einzelne Zentren) umgesetzt sind [12]. Aus den Schilderungen der Teilnehmenden aus den PAIN2020-Zentren lässt sich entnehmen, dass diese Empfehlungen sowohl auf die Anwendung der in PAIN2020 avisierten Zielgruppe als auch auf ambulant organisierte Umsetzungen auszuweiten und einzuhalten sind [15]. Ein erlebter Mangel aus Sicht der beteiligten Berufsgruppen besteht vor allem aber in der bisher kaum ausgestalteten inhaltlichen Umsetzung, die aber die tägliche Arbeit mit den Patient:innen maßgeblich prägt. Hier wurde der Wunsch deutlich, entsprechend passende und weiterführende Konzepte zu entwickeln.

Die Ergebnisse des Workshops weisen über die Struktur- und Prozessparameter hinaus auf bisher weniger beachtete Aspekte der interdisziplinären Zusammenarbeit hin, die relevante Potenziale für die inhaltliche und qualitative Weiterentwicklung bieten. Darunter fallen insbesondere die Bereiche der personalen Kompetenz (Selbstständigkeit, Sozialkompetenz), Methoden- und Fachkompetenz (Wissen und Fertigkeiten) sowie die Qualifikation und Erfahrung (z. B. Routine oder Häufigkeit der Zusammenarbeit) in Teams, die Einfluss auf die interdisziplinäre Zusammenarbeit haben. Daraus leiten sich verschiedene Aspekte ab, die zukünftig Berücksichtigung finden sollten:

- Identifikation von Basiskompetenzen für interdisziplinäre Zusammenarbeit,
- Identifikation von Gesprächsführungskompetenzen zur Moderation von Teamgesprächen,
- Erarbeitung eines konstruktiven und transparenten Rollenverständnisses der drei Berufsgruppen untereinander,
- Thematisieren der Grenzen der eigenen Berufsgruppen *und*
- Schaffen einer Feedbackkultur in Teams/einer wertschätzenden Haltung im Team.

### Limitationen

Das gewählte Format des World Cafés und die interdisziplinäre Zusammensetzung der Gruppen boten einerseits eine gute Struktur zur Bearbeitung der gesetzten Fragestellungen, sie boten aber auch eine gewisse Flexibilität in der Anwendung, wenn Inhalte von den Teilnehmenden selbst weiterentwickelt werden wollten. Insbesondere durch die rotierenden Durchgänge und die interdisziplinäre Zusammensetzung der Gruppen entstanden immer wieder neue (vertiefende) Perspektiven auf die Frage der Gestaltung von Interaktion, das Einbringen von Erfahrungen aus dem Versorgungsalltag sowie eine Bestätigung oder „Verwerfung“ von Inhalten. Ein weiterer, aus unserer Sicht limitierender Faktor besteht in der begrenzten Teilnehmendenzahl. Die Ergebnisse können demnach nicht als erschöpfend im Sinne einer Sättigung des Datenmaterials angesehen werden.

Wie oben bereits dargelegt, kann das vorgestellte Rahmenmodell als eine erste, vorläufige Diskussionsgrundlage angesehen werden, es stellt einen Anfang dar. Es dient einer ersten Orientierung, in welcher Weise interprofessionelle Teamzusammenarbeit weiterentwickelt werden könnte. Auch hier ist eine Erschöpfung der Kriterien, Aspekte und Inhalte nicht anzunehmen. Für eine vertiefte Bearbeitung wären repräsentative Untersuchungen wünschenswert, die zukünftig auch in qualitätssichernde Maßnahmen und klinische Untersuchungen zum Therapieoutcome bzw. der Patient:innen- und Mitarbeiter:innenzufriedenheit in interdisziplinären Versorgungsangeboten für Schmerztherapie münden sollten.

Die beschriebenen Einschränkungen werden jedoch aus unserer Sicht dadurch aufgehoben, dass wir einen intensiven, neuartigen Einblick in die Belange integrativer, interprofessioneller und interdisziplinärer Teamarbeit aus Sicht der Praktiker:innen erhalten haben, den es unserer Kenntnis nach so für die Schmerztherapie noch nicht gibt. Unter der Voraussetzung, die von uns präsentierten Ergebnisse primär als explorativ zu verstehen, kann das Rahmenmodell Anregungen und Impulse für weitere konzeptuelle Entwicklungsarbeit und wissenschaftliche Untersuchungen bieten. Dabei stehen aus unserer Sicht neue Dimensionen bzw. Entwicklungsfelder (u. a. [Basis-]Kompetenzen, Gesprächsführung/Moderation in interdisziplinären Teams, Konfliktlösung etc.) zur Diskussion. Diese könnten wiederum Grundlagen für die Entwicklung entsprechender (interprofessioneller) Schulungskonzepte sowie Mitarbeiter:innenprofile und -qualifikationsanforderungen sein. Darüber hinaus sehen wir zunehmend auch Ansatzpunkte für evidenzbasierte Konzepte zur inhaltlichen Ausgestaltung der Arbeit mit den Patient:innen (bspw. *shared decision making*), die in der weiteren Diskussion hochqualitativer Umsetzung von interdisziplinären Ansätzen nicht nur für Patient:innen mit wiederkehrenden Schmerzen und Chronifizierungsrisiko gelten mögen.

## Fazit für die Praxis

Die Ergebnisse der Arbeit geben einen Einblick in die Interaktion der Berufsgruppen untereinander bzw., wenn auch nachgeordnet, mit den Patient:innen im Rahmen der Teamsitzung und des Abschlussgesprächs im IMA in PAIN2020. Diese entstanden durch Erfahrungen in der klinischen Anwendung und sind aus unserer Sicht somit für die Umsetzung im Versorgungsalltag relevant. Für die Umsetzung des IMAs in der Versorgung traten interaktionelle Fragen in den Vordergrund. Als Grundvoraussetzungen für eine Weiterentwicklung gelungener interdisziplinärer Zusammenarbeit ergeben sich aus Sicht der Autor:innen daher zwei erste Schritte: 1. Die Berücksichtigung von personalen Kern-Kompetenzen und Fachkompetenzen (u. a. Gesprächsführung/Moderation) für eine erfolgreiche interdisziplinäre Zusammenarbeit neben den bereits etablierten Prozess- und Strukturparametern in der Definition von Rahmenbedingungen für die IMST. 2. Die Berücksichtigung von interaktionsfördernden Aspekten in der Gestaltung von Schulungsangeboten, wie z. B. professionsübergreifende Module, zu Regeln einer förderlichen Feedbackkultur sowie zur sanften Auseinandersetzung mit Rollenbildern und -verständnis im Team als Vorbereitung auf einen gleichberechtigten Austausch.
